# Solitary rectal ulcer syndrome: A systematic review: Erratum

**DOI:** 10.1097/MD.0000000000014662

**Published:** 2019-02-15

The article, “Solitary rectal ulcer syndrome: A systematic review”,^[1]^ which appeared in Volume 97, Issue 18 of *Medicine*, was published under the incorrect article type and should be published as a study protocol.

The correct title is “Solitary rectal ulcer syndrome: A systematic review and Meta-Analysis Study Protocol.”
